# Effectiveness of Ureteroscopy-Assisted Retrograde Nephrostomy (UARN) for Percutaneous Nephrolithotomy (PCNL)

**DOI:** 10.1371/journal.pone.0052149

**Published:** 2012-12-14

**Authors:** Takashi Kawahara, Hiroki Ito, Hideyuki Terao, Yoshitake Kato, Hiroji Uemura, Yoshinobu Kubota, Junichi Matsuzaki

**Affiliations:** 1 Department of Urology, Ohguchi Higashi General Hospital, Yokohama, Kanagawa, Japan; 2 Department of Urology, Yokohama City University Graduate School of Medicine, Yokohama, Kanagawa, Japan; University of Colorado, United States of America

## Abstract

**Objective:**

To determine the impact of ureteroscopy-assisted retrograde nephrostomy (UARN) during percutaneous nephrolithotomy (PCNL).

**Materials and Methods:**

From April 2009 to September 2011, a total of 50 patients underwent PCNL for large renal stones (stone burden >2 cm). We performed UARN in the Galdakao-modified Valdivia position for 27 patients (UARN PCNL) and ultrasonography-assisted percutaneous nephrostomy in the prone position for 23 patients (prone PCNL).

**Results:**

UARN PCNL significantly improved the stone-free rate (81.5% vs 52.2%) and the rate of residual stones (<4 mm, 92.6% vs 65.2%, P<0.05). The median length of the operation was significantly shorter for UARN PCNL, at 160 min, compared to 299 min for prone PCNL (P<0.001). There was one intraoperative complication in prone PCNL, namely a hemorrhage that resulted in stopping the initial treatment, but it was cured conservatively. The postoperative complications included a high grade fever that persisted for three days in two UARN PCNL patients (7.4%) and six prone PCNL patients (26.1%). The Clavien grading scores showed significantly lower postoperative complications for UARN PCNL compared to prone PCNL.

**Conclusion:**

UARN is associated with a higher stone-free rate, shorter operation time, and fewer complications during PCNL than prone PCNL.

## Introduction

Goodwin *et* *al*. first reported the percutaneous renal access in 1955 [1]. After that, the percutaneous nephrolithotomy (PCNL) technique was developed, and PCNL became the standard procedure for large renal stones. Ultrasound-guided puncture of the renal collecting system with subsequent placement of a drainage tube under fluoroscopic guidance is currently the standard modality for percutaneous nephrostomy.

Even when percutaneous access is successfully gained with a needle puncture in the non-dilated collecting system, the tract is often not in the most desirable location for stone extraction [2]. To resolve this problem, retrograde nephrostomy was first developed by Lawson *et* *al*. in 1983, and Hunter *et* *al*. reported 30 cases of retrograde nephrostomy in 1985 [2], [3]. With this procedure, after the needle has exited through the skin, no further steps are required in preparation for dilation.

We previously described ureteroscopy (URS)-assisted retrograde nephrostomy (UARN) [4], [5]. In UARN, it is possible to continuously visualize the dilation from puncture to insertion of the nephroaccess sheath (NAS). This study examined the impact of UARN during PCNL on the stone free-rate, rate of residual stones <4 mm, length of the operation, reoperative risk, and complications for patients with large renal stones (stone burden >2.0 cm).

## Patients and Methods

### Patient characteristics

From April 2009 to September 2011, a total of 50 patients underwent PCNL for large renal stones (stone burden >2 cm). We performed UARN in the Galdakao-modified Valdivia position for 27 patients (UARN PCNL group) and ultrasonography (US)-assisted nephrostomy in the prone position for 23 patients (prone PCNL group). This study was approved by the Institutional Review Board of Ohguchi Higashi General Hospital. Written informed consent was obtained from all patients. All patients underwent PCNL at Ohguchi Higashi General Hospital by the same surgical group (TK, HI, HT, JM).

Prone PCNL were performed between April 2009 and March 2011 and UARN PCNL was performed between August 2010 and September 2011. Between August 2010 and March 2011, which was a period of transition from prone PCNL to UARN PCNL, prone PCNL was performed in five cases and UARN PCNL was performed in four cases. The exclusion criteria included having undergone either nephrostomy or shockwave lithotomy (SWL), URS, or PCNL previously.

### Preoperative analysis

The patients' stone burden was defined as the sum of the maximum stone diameters, and the maximum stone size was determined by the long axis of the largest stone by imaging on kidney-ureter-bladder (KUB) films and computed tomography (CT) with or without intravenous urography (IVU). We usually performed pre-procedural urine cultures, however, we did not change the antibiotics based on the results of the urine cultures in this study, because a positive urine culture does not always reflect the bacteria which would cause a urinary tract infection in that patient. We administered pre- and post-procedural cefotiam for four days to all patients.

### Ultrasonography-assisted nephrostomy with fluoroscopic guidance

Under general and epidural anesthesia, the patient was placed in the lithotomy position. After cystoscopy and retrograde ureteric catheterization, using an occlusion balloon catheter, the patient was placed in the prone position. Selective calyceal puncture, usually in the lower calyx, was carried out with an 18 gauge needle (Boston Scientific, MA, USA) under ultrasonographic assistance with fluoroscopy. A guidewire was placed into the ureter, then a 24 Fr or 30 Fr percutaneous NAS (X-Force®N30 Nephrostomy Balloon Dilation Catheter, BARD, NJ, USA) was passed over the balloon into the calyx under fluoroscopic guidance, and the balloon was removed.

### Ureteroscopy-assisted retrograde nephrostomy (UARN)

**Figure 1:**
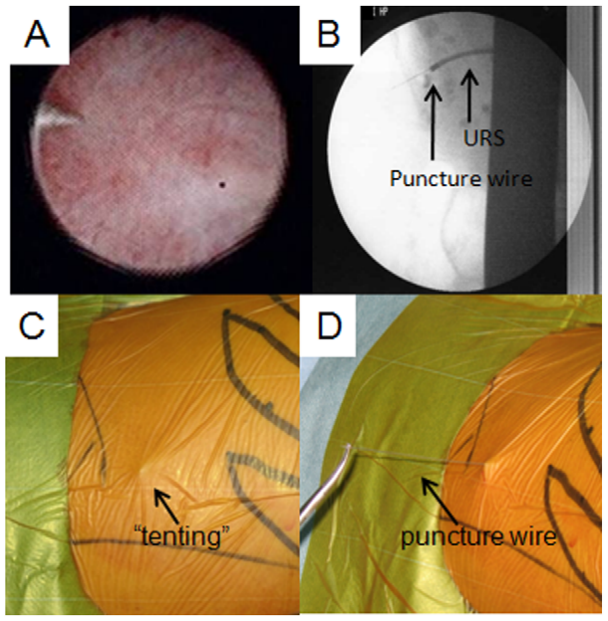
The technique of ureteroscopy assisted retrograde nephrostomy (UARN). Puncturing under (A) ureteroscopy and (B) fluoroscopic guidance. (C) Tenting at the posterior axillary line. (D) Grasping the puncture guidewire.

Under general and epidural anesthesia, the patient was placed in the Galdakao-modified Valdivia position [6], [7], [8], [9] [10]. A 6 Fr rigid ureteroscope (Uretero-Renoscope®, Karl Storz, Tuttlingen, Germany) was advanced; if it did not encounter either ureteral stenosis or ureteral stones, a flexible ureteroscope (Flex-X^2^®, Karl Storz, Tuttlingen, Germany) was inserted through a 12/14 Fr (inner/outer) 35 cm ureteral access sheath (UAS) (Flexor®, COOK Urological, IN, USA) or a 11/13 Fr 46 cm UAS (Navigator® 11 Fr 46 cm, Boston Scientific, MA, USA) to the ureter. We carefully observed the target calculi and defined the appropriate renal calyx to puncture. Thereafter, a Lawson retrograde nephrostomy puncture wire (Lawson Retrograde Nephrostomy Wire Puncture Set, COOK Urological, IN, USA) was carefully inserted into the flexible ureteroscope [2]. The flexible ureteroscope approached the desired renal calyx again, and the route from the renal calyx to exiting the skin was then confirmed under fluoroscopy [Figs. 1A and B].

**Figure 2:**
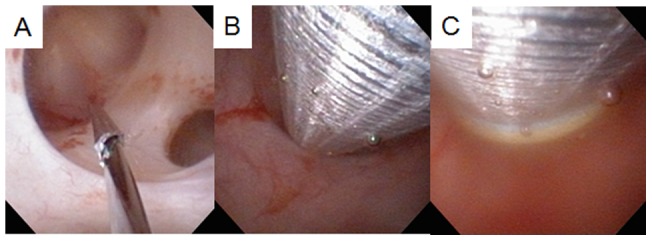
Dilating the nephrostomy is under visualization with ureteroscopy. (A) Needle dilation. (B) Balloon dilation. (C) Inserting the NAS.

To avoid injury to the spleen, liver, intestines, or pleural cavity, the puncture was performed under ultrasonography after more than two urologists rechecked the preoperative CT. Ultrasonography confirmed that there were no organs surrounding the area from the root from the URS to the skin, and in case another puncture spot needed to be chosen, we rechecked the root using ultrasonography. The puncture wire was passed through the muscle easily and “tented” the skin at the posterior axillary line [Fig. 1C]. The skin was incised and the needle was delivered [Fig. 1D]. Next, the 22 G and 18 G needle dilator were placed over the puncture wire, which was advanced through the skin, subcutaneous fat, abdominal wall musculature, and perinephric fat until it reached the renal parenchyma [Fig. 2A]. After the catheter dilation up to 12 Fr was performed, a safety guidewire was placed through the UAS. A 24 Fr or 30 Fr percutaneous NAS (X-Force®N30 Nephrostomy Balloon Dilation Catheter, BARD, NJ, USA) was passed over the balloon into the calyx under ureteroscopic and fluoroscopic guidance, and then the balloon was removed [Figs. 2B and C].

### Percutaneous nephrolithotomy (PCNL)

Calculus fragmentation was undertaken using the Swiss LithoClast® pneumatic lithotripter (EMS, Nyon, Switzerland) through a rigid nephroscope (percutaneous nephroscope, KarlStorz, Tuttlingen, Germany). We had already optimized our procedures (the duration of litholysis was limited to around 1.5 hr) to decrease the risk of complications such as systemic infection, deep venous thrombosis, complications related to anesthesia, nerve palsy, and rhabdomyolysis [11]. If residual stone fragments were observed at the end of the initial treatment or on postoperative KUB the following day, residual stone fragments were removed seven days after the initial treatment with URS-assisted PCNL in the Galdakao-modified Valdivia position in both groups. Small stone fragments in any calyx where the nephroscope could not reach were lysed using a Ho: YAG laser (Versa Pulse Power Suite 100W® or Versa Pulse 30W®, LUMENIS Surgical, CA, USA) using a flexible ureteroscope or flexible cystoscope (Olympus, Tokyo, Japan). The nitinol stone retrieval baskets used included the 1.5 Fr N-circle® (Cook Urological, IN, USA) and 1.9 Fr Zerotip® (Boston Scientific, MA, USA). Ureteral stents were placed at the conclusion of all ureteroscopic procedures. Patients whose vital signs were stable and who had no intraoperative complications had the wire removed and the wound compressed for three min.

### Postoperative analysis

A stone-free outcome was defined as total fragmentation of the stone(s) to 0 mm, as detected by imaging on KUB films and CT with or without IVU at a follow-up performed 6 to 8 weeks postoperatively. Residual stones were detected by imaging on KUB and CT with or without IVU within this follow-up period. Completed procedures during the initial treatment were defined as those in which the patients did not require a second treatment with URS-assisted PCNL by postoperative day 7 or additional treatment (SWL, URS, and PCNL) for the target stone. In most cases, residual renal stone fragments >1 cm and ureter stones, regardless of size, were recommended for additional treatment. The final decision to proceed with additional treatment was made cooperatively by the surgeons and patients.

### Complications

Perioperative complications were assessed and scored according to the modified Clavien classification system as applied to PCNL [12], [13], [14]. We defined fever as a body temperature >38.5°C for more than three days.

### Statistical analysis

All continuous variables are expressed as the means ± SD. The numerical data were compared by Student's *t*-test. The differences between two groups were compared by one-factor ANOVA. A *P* value ≤0.05 was considered to be significant.

## Results

Fifty patients were included in this study (27 in the UARN PCNL group and 23 in the prone PCNL group) who had a renal stone burden of >2.0 cm. All stone locations were renal. The median stone burden was 57 mm (mean 59.1±28.1 mm) in the UARN PCNL group and 51 mm (mean 57.6±20.7 mm) in the prone PCNL group. The median maximum stone size was 32 mm (mean 32.7±13.5 mm) in the UARN PCNL group and 37 mm (mean 38.2±13.2 mm) in the prone PCNL group. The patients' characteristics, including age, affected side, location, and number of stones are shown in Table 1. In this study, one patient had a horseshoe kidney and one obese patient who had been previously described in a case report, were also included in this study [15].

**Table 1:** The patient characteristics.

Variables	No. (%) or Median (Mean ± SD)		*P* value
	UARN PCNL	Prone PCNL	
No. of patients	27	23	
Median age (years)	58 (57.2±12.3)	57 (55.5±11.4)	n.s.
Sex			
Male (%)	16 (59.3%)	11 (47.8%)	n.s.
Female (%)	11 (40.7%)	12 (52.2%)	
Side			
Right (%)	8 (29.6%)	11 (47.8%)	n.s.
Left (%)	19 (70.4%)	12 (52.2%)	
Stone burden (mm)	57 (59.1±28.1)	51 (57.6±20.7)	n.s.
Maximum stone size (mm)	32 (32.7±13.5)	37 (38.2±13.2)	n.s.
Mean CT density (HU)	1397 (1343±63.8)	1352 (1317±62.0)	n.s.
No. of stones	3 (3.9±2.9)	3 (3.6±3.3)	n.s.
Solitary	6 (22.2%)	6 (26.1%)	n.s.
2 or 3	9 (37.3%)	10 (43.5%)	
≥4	11 (40.7%)	7 (30.4%)	

n.s.: not significant, UARN: URS assisted retrograde nephrostomy, PCNL: percutaneous nephrolithotomy.

Table 2 shows the intraoperative and postoperative outcomes. UARN PCNL significantly improved the stone-free rate and rate of residual stones <4 mm (*P* = 0.027 and *P* = 0.015, respectively). The median length of the operation was significantly shorter for UARN PCNL, at 160 min (mean 187.1±77.4 min), compared to 299 min (mean 297.5±100.4 min) for prone PCNL. Completion during the initial treatment was achieved in 17 of the 27 patients (62.9%) in the UARN PCNL group and 8 of the 23 patients (34.8%) in the prone PCNL group. The UARN PCNL group therefore showed a higher rate of completion during the initial treatment, although the difference was not significant (*P* = 0.048). A stone analysis showed no differences between the two groups.

**Table 2:** The intra- and postoperative clinical outcome.

Variables	Number (%) or Median (Mean ± SD)		*P* value
	UARN PCNL	Prone PCNL	
No. of patients	27	23	
Stone free (0 mm)	22 (81.5%)	12 (52.2%)	0.027
Residual stones (<4mm)	25 (92.%)	15 (65.2%)	0.015
Completed during initial operation	17 (62.9%)	8 (34.8%)	0.048
Fever (>38.5°C for 3 days)	2 (7.4%)	6 (26.1%)	0.075
Clavien grading scores			
0	24 (88.9%)	13 (56.5%)	0.005
I	3 (11.1%)	7 (30.4%)	
II	0 (0%)	3 (13.0%)	
≥III	0 (0%)	0 (0%)	
Stone analysis			
Calcium oxalate	12 (44.4%)	10 (43.5%)	n.s.
Calcium oxalate and calcium phosphate	9 (33.3%)	7 (30.4%)	
Calcium phosphate	3 (11.1%)	0 (0.0%)	
Calcium phosphate and uric acid	0 (0.0%)	2 (8.7%)	
Struvite	1 (3.7%)	0 (0.0%)	
Struvite and calcium phosphate	1 (3.7%)	2 (8.7%)	
Uric acid	1 (3.7%)	1 (4.3%)	
Unknown	0 (0.0%)	1 (4.3%)	
Total length of operation (min.)	160 (187.1±77.4)	299 (297.5±100.4)	<0.001

There was one intraoperative complication in the prone PCNL group, which was a hemorrhage resulting in stopping the initial treatment, but it was cured conservatively. None of the patients required a blood transfusion. The postoperative complications included a high grade fever that persisted for three days in two patients (7.4%) in the UARN PCNL group and six patients (26.1%) in the prone PCNL group. None of the UARN PCNL patients had Clavien grading scores ≥3. On the other hand, three patients in the prone PCNL group had Clavien grading scores of 2, including two cases of urosepsis and one of colon injury, which were cured conservatively.

In two cases, the ureteroscope could not reach to the target calyx because a renal stone occupied the ureteropelvic junction. We advanced the tip to the target calyx using Ho: YAG laser lithotripsy passing through the ureteroscope, then UARN was performed. In these cases, because of the dilated collecting systems, US-guided nephrostomy might have been possible, but even if it took more time to perform litholysis, puncturing and dilating the nephrostomy under visualization contributed to the safety and effectiveness of the operation, making it the ideal position for nephrostomy. The times from litholysis to passing through the ureteroscope were 4.0 min and 5.2 min, respectively.

The present study included two patients whose renal stones were in the lower calyx of a horseshoe kidney [15]. Some patients with this condition have been successfully treated with URS, although due to the altered anatomical relationships in this disorder, ureteroscopic approaches can be challenging and are not universally recommended [16], [17]. PCNL is therefore considered to be suitable for treating renal calculi in the lower calyx in horseshoe kidneys. To reach the lower calyx, a nephrostomy is usually created in the upper calyx [18], because it is difficult to avoid injuring the surrounding organs while performing nephrostomy on the target calyx. UARN provides two advantages in approaching a horseshoe kidney. First, it allows for confirmation of whether the ureteroscope can reach the target stone in the lower calyx or not; second, under UARN, it is possible to identify the target calyx and the route from the calyx to the skin under fluoroscopic guidance and adjust the angle continuously.

This study also included two obese patients, with body mass indices of 33.0 and 34.0 kg/m^2^, respectively. In both cases, the adequacy of the puncture and the ease of changing direction without extracting the puncture wire from the ureter were easily obtained with UARN.

UARN was performed in one case of complete staghorn calculi. During surgery for renal staghorn calculi with no hydronephrosis, percutaneous nephrostomy is sometimes difficult even when a balloon occlusion catheter is used to dilate the renal collecting system [19]. In this case, the calculus occupied the entire renal calyx, so we speculated that advancing the guidewire to the ureter before dilation would be difficult even if percutaneous nephrostomy succeeded using US or fluoroscopy. We successfully performed UARN from the dorsal side of the renal pelvis and also completed PCNL. We also experienced one case of a functional solitary kidney, which was successfully treated by UARN PCNL.

## Discussion

Although there is a limitation associated with our study, due to the fact that this was a case controlled study and not a randomized study, UARN PCNL showed higher stone free rates and fewer complications compared to the Prone PCNL. PCNL has become the preferred method for treating patients with a large or complex stone burden [20], [21]. Ultrasound-guided puncture of the renal collecting system with subsequent placement of a drainage tube under fluoroscopic guidance is currently the standard modality for percutaneous nephrostomy. Although it helps to avoid a puncture of the liver or spleen, this method cannot reliably visualize air-filled structures, such as the bowel [22]. Inserting the guidewire into the ureter is safe and effective for dilating and inserting the NAS; however, in nondilated renal collecting systems, as in staghorn calculi, inserting the guidewire into the ureter is very difficult.

Our procedure is performed in the Galdakao-modified Valdivia position. In 1987, Valdivia-Uria described a percutaneous nephrolithotomy with the patient in supine position, with a 3-L serum bag placed below the flank [6]. Both surgical and anesthesiological advantages were described to result from using this position. In 2001, Ibarluzea *et* *al.* reported a Galdakao–modified Valdivia position [10], in which the supine position was the same as in the Valdivia position, but the leg of the target side was extended, while the contralateral one was abducted; this position had the advantage of allowing simultaneous percutaneous and retrograde access [23]. In the present study, we continuously visualized the motion of the ureteroscope easily under ultrasonography, and were able to detect the tent sign easily without changing the patient's position.

Retrograde nephrostomy was first developed by Lawson et al. in 1983, and Hunter et al. reported 30 cases of retrograde nephrostomy in 1985 [2], [24]. A Lawson retrograde nephrostomy wire puncture set (COOK Urological, USA) is now easily obtained. In addition, several publications using this device have recently been reported, including our report [4], [25]. In this procedure, after the needle has exited through the skin, no further steps are required in preparation for dilation, and UARN provides continuous visualization from puncture to PCNL, including the needle, catheter, and balloon dilation and insertion of the NAS. The UARN technique is based on URS, not PCNL. In this study, a total of four surgeons performed this procedure. We believe that more than 50 experiences with URS are sufficient for overcoming the learning curve associated with this procedure. Because this procedure contributes to continuous visualization, no highly difficult techniques are needed, such as blind techniques.

All patients were able to undergo UARN in this study. At our institute, a total of 97 patients were scheduled to undergo UARN until September 2012. Of these, 10 patients did not undergo the planned procedure. Two patients were not treated by UARN due to safety concerns when the tent sign seemed to be on the ventral side. Five patients were not treated by UARN due to the intraoperative findings. These patients had large ureter stones, so the surgeon decided to perform the puncture percutaneously to avoid increasing the intrarenal pressure. In the other three cases, the renal capsule was so hard that the puncture wire could not pass through to the renal capsule. These patients also had severe urinary tract infections preoperatively.

The Clinical Research Office of the Endourological Society (CROES) was established in 2008, and the PCNL Global Study included more than 5800 patients from 26 countries [26]. In that study, while the length of the operation and stone-free rates were in favor of prone PCNL, lower rates of patient morbidity favored supine PCNL [27]. In contrast, our data showed a higher stone-free rate and shorter operation for UARN PCNL than prone PCNL. With respect to the length of the operation, under UARN, after the needle has exited the skin, no further steps are required in preparation for dilation. Moreover, dilation and insertion of the NAS is continuously visualized, so no adjusting of the position of the NAS is needed. The Galdakao-modified Valdivia position facilitates both percutaneous and transurethral approaches, results in a shortened operation because no position changes from the lithotomy position to prone (and vice versa) are necessary. In this study, both the stone-free rate and the rate of residual stones <4 mm were significantly higher in UARN PCNL than in prone PCNL. We speculate that this is because UARN facilitates continuous visualization of the URS, so ideal puncture of the calyx is easily performed, and results in ideal NAS insertion in front of the target stone. The rate of completed initial treatment in UARN PCNL was higher than that in prone PCNL, although not significantly so (*P* = 0.048).

Litholysis was stopped in one patient (4.3%) in the initial treatment in the present study because of hemorrhage. None of the patients received a blood transfusion. A postoperative fever (body temperature >38.5°C lasting >3 days) was observed in two patients (7.4%) in the UARN PCNL group and six patients (26.1%) in the prone PCNL group. With regard to the fever, our rate of fever in the prone PCNL group may have been slightly higher than that in the previous global studies [26]. However, compared to the data reported in the CROES global study, our patients had a larger stone burden. Therefore, further studies are needed to accurately determine the risk of fever in typical patients. The Clavien grading score in the CROES showed Grade 1 in 11.1%, Grade 2 in 5.3% and Grade 3 in 2.3% of cases. Our results in UARN PCNL were not inferior to the CORES study (11.1% in Grade 1 and 0% in Grade 2 or more), while our patients who underwent prone PCNL showed inferior conditions compared to the patients in the CORES study (30.4% in Grade 1 and 13.0% in Grade 2). This might also be explained by the patients' large stones. However, further studies are needed to compare the complications associated with UARN PCNL to the standard PCNL.

Residual stones were found in the lower calyx in three patients. Two patients did not want an additional tract for the residual stone in the lower calyx, and in one patient, an additional tract was made under fluoroscopic guidance for retracting the residual stones. In that case, URS also supported visualization from puncture to the insertion of the NAS. The disadvantage of UARN was that it was difficult to reach the lower calyx: the puncture wire cannot traverse the tight bend in the ureteroscope necessary to address the lower calyx. Therefore, to puncture the lower calyx, it is more suitable to place the puncture wire outside the ureteroscope or create a nephrostomy under ultrasonographic or fluoroscopic guidance. However, in such cases, the ureteroscope also supports the visualization of the insertion and dilation of the nephrostomy.

We did not assess the total cost in these two groups. However, the rate of completion in the initial operation in the UARN group was 62.9%, while it was 34.8% in the prone PCNL group. The rest of the patients needed a second session URS seven days after the initial PCNL. Under the Japanese insurance system, the procedure cost of PCNL is 328,000JPY (about 4,000USD) and the cost of URS is 222,700JPY (about 2,800USD). Therefore, we believe that there may also be a better cost effectiveness for UARN due to the higher rate of completion during the initial procedure.

As noted above, the major limitation of this study was that it was not a randomized double blind study. The two methods of treatment were asynchronously adopted in two different time intervals. Therefore, although we recommended that patients undergo the procedures based on the EAU or AUA guidelines (more than 2 cm stones), a patient selection bias was still present. Further studies are needed to confirm the effectiveness of UARN PCNL compared to the standard technique.

## Conclusions

UARN was thus found to obtain a higher stone-free rate, shorter operation time, and fewer complications during PCNL than does prone PCNL.
